# Effectiveness and functional magnetic resonance imaging outcomes of Tuina therapy in patients with post-stroke depression: A randomized controlled trial

**DOI:** 10.3389/fpsyt.2022.923721

**Published:** 2022-06-30

**Authors:** Jiming Tao, Shuaipan Zhang, Lingjun Kong, Qingguang Zhu, Chongjie Yao, Qingjuan Guo, Jiajia Wu, Chunlei Shan, Min Fang

**Affiliations:** ^1^Yueyang Hospital of Integrated Traditional Chinese and Western Medicine, Shanghai University of Traditional Chinese Medicine, Shanghai, China; ^2^School of Acupuncture-Moxibustion and Tuina, Shanghai University of Traditional Chinese Medicine, Shanghai, China; ^3^Institute of Tuina, Shanghai Institute of Traditional Chinese Medicine, Shanghai, China; ^4^Shuguang Hospital, Shanghai University of Traditional Chinese Medicine, Shanghai, China

**Keywords:** Tuina, traditional Chinese medicine, functional magnetic resonance imaging, post-stroke depression, cognitive function

## Abstract

**Objective:**

To investigate the effectiveness and functional magnetic resonance imaging (fMRI) outcomes of Tuina therapy in patients with post-stroke depression (PSD).

**Methods:**

This was a single-center, randomized, two-armed, controlled trial. Eighty-four patients with PSD were selected and randomly assigned to a Tuina therapy group or a routine rehabilitation control group. The patients underwent five 20-min treatment sessions per week over a period of 2 weeks. The primary outcome measure was change in Hamilton Depression Rating Scale (HAMD) score over the 2 weeks of intervention, whereas the secondary outcome measures were changes in Fugl-Meyer Assessment (FMA) score, Modified Barthel index (MBI), and Mini Mental State Examination (MMSE) score.

**Results:**

The Tuina group showed significantly improved HAMD scores compared to the routine rehabilitation control group (5.85, [2.54, 9.16]). For the secondary outcomes, the Tuina group showed better MMSE scores than the routine rehabilitation group (1.97, [1.19, 2.76]); however, there were no significant differences between the other secondary outcomes of both groups (*P* > 0.05). After 2 weeks, both groups showed a significant decrease in HAMD score compared to baseline. In addition, the Tuina group showed a significant decrease in MMSE score compared to baseline (2.35, [1.8, 2.9]); however, there were no significant differences in the MBI and FMA scores of the two group after the intervention (*P* > 0.05). Regarding fMRI results, the zALFF values of the right caudate nucleus, right putamen, right insula, left superior temporal gyrus, right parahippocampal gyrus, right hippocampus, left middle temporal gyrus, left angular gyrus, and left thalamus were higher in the Tuina group. In the Tuina group, the functional connectivity between the hippocampus and thalamus, and the thalamus and caudate nucleus, were significantly different (*P* <0.01). In addition, the zALFF value of the hippocampus was significantly negatively correlated with HAMD score. No serious adverse events were observed in both groups.

**Conclusion:**

Tuina therapy administered 10 times within 2 weeks is safe and can effectively relieve depression and improve cognitive function in patients with PSD. This finding may be closely related to the effect of Tuina therapy on the activation and functional connectivity of the hippocampus.

**Clinical Trial Registration:**

http://www.chictr.org.cn/showproj.aspx?proj=55151, identifier ChiCTR200003388.

## Introduction

Post-stroke depression (PSD) is caused by cerebrovascular organic disease, and is a common complication after stroke, mainly manifesting as depression, sleep disturbance, and social withdrawal. PSD negatively affects the activities of daily living (ADL) and cognitive function of patients with stroke (1, 2). In addition to depression, impaired body and cognition functions, which are prominent clinical symptoms, are long-term effects of PSD (3). Negative cognition, motor dysfunction, and poor ADL interact with each other in the pathogenesis of PSD, and their specific recovery mechanism had become an important target for PSD research.

A previous meta-analysis showed that the PSD occurs in approximately one-third of all stroke cases, with a higher incidence among patients with acute stroke (4). PSD places a heavy burden on families and society and seriously hinders the functional recovery of stroke survivors (5). In addition, PSD is an important predictor of all-cause mortality after stroke. At present, a long-term follow-up study on PSD, conducted by a multidisciplinary team, is ongoing (6, 7). Some clinical studies have proven that antidepressants are more effective than placebo for the treatment of PSD. However, the clinical efficacy and specificity of various antidepressants cannot be accurately evaluated in evidence-based studies. In addition, it has been confirmed that some antidepressants are associated with stroke recurrence (8–11).

Furthermore, although clinical guidelines recommend pharmacotherapy as the preferred option for the treatment of PSD, its use is limited by variability in symptoms and side effects (10). Rehabilitation is a process of positive change that involves the use of knowledge and skills to achieve optimal physical, mental, and social functioning (12). Routine rehabilitation, including the Brunnstrom Bobath, and proprioceptive neuromuscular facilitation therapies, which are mainly based on guided joint and separation movements for the improvement of physical dysfunction to achieve psychological modulation, is widely used for the treatment of patients with stroke (13, 14). In China, Tuina therapy, a traditional Chinese qigong exercise-based alternative manual therapy used for improving depression and anxiety, has been used for the rehabilitation for patients with stroke (15–17). Tuina therapy involves pressing and kneading the acupoints on the posterior midline of the spine using the fingers, and rubbing along the Du meridian on the spine using the palms. The herbal ointment used for Tuina therapy consists of steam-distilled essential oils extracted from Chinese herbal medicines, petrolatum, and glycerin, and is applied on the back (18).

Functional magnetic resonance imaging (fMRI) scans of patients with PSD show decreased excitability in the ipsilateral side of the brain region with stroke lesions, accompanied by pathological features of cortical and subcortical fiber injury. Previous brain fMRI studies have demonstrated that Tuina may stimulate certain cortical excitability and connectivity changes in the higher centers of the brain, which exert antidepressant effects (19, 20). However, there is no evidence of the efficacy of Tuina for the treatment of PSD. In addition, the neuroimaging mechanisms of the effects of Tuina in patients with PSD are unclear. Therefore, the aim of this study was to evaluate the efficacy and characteristic resting-state fMRI outcomes of Tuina therapy for the treatment of PSD.

## Materials and methods

### Study design

This was a double-arm, parallel, randomized controlled trial of the curative effect of Tuina on PSD compared to routine rehabilitation therapy. The protocol of this trial was approved by the Ethics Committee of Yueyang Hospital of Integrated Traditional Chinese and Western Medicine affiliated to Shanghai University of Traditional Chinese Medicine (project number: 2020-014). Informed verbal and written consent were obtained from all the patients. Patients were informed of the study design and were randomly assigned to a Tuina group or a routine rehabilitation group in a 1:1 ratio.

### Participant recruitment

Patients were recruited from the departments of rehabilitation in Yueyang Hospital of Integrated Traditional Chinese and Western Medicine affiliated to Shanghai University of Traditional Chinese Medicine from September 2020 to December 2021. Patients were recruited based on the standard diagnostic criteria for PSD in the Diagnostic and Statistical Manual of Mental Disorders (III, IV, or Vth edition) (9). The inclusion criteria were as follows: (1) aged between 20 and 80 years old, regardless of sex; (2) scored 7 or higher and 24 or less in the first 17 categories of the Hamilton Depression Rating Scale (HAMD); (3) willingness to participate in the trial and can tolerate fMRI assessment; (4) can understand and follow the trial protocol with right-handed; (5) TOAST stroke subtype is large arteriosclerosis type, small artery occlusion type, or cerebral infarction; and (6) the damage site is the non-frontal lobe, parietal lobe, temporal lobe, or other cerebral infarction cortical areas. Patients with the following conditions were excluded: (1) a high risk of attempting suicide; (2) severe intellectual impairment, language impairment, or mental disorder; (3) severe adverse reactions to antidepressants; (4) uncomfortable reaction to Tuina; (4) acute (within 2 weeks) or progressive stage of stroke with unstable vital signs; (6) non-magnetic metal objects near the head or in the brain, including cochlear implants and metal glasses; and (7) restricted or lacks the capacity for civil conduct.

### Randomization and blinding

Randomly selected sequences were sealed in opaque envelopes and given to individuals unaffiliated with the trial for the assignment of the participants. Owing to the nature of physical therapy, the participants could not be blinded to the intervention; however, the assessors and data analysts were blinded to the group assignments.

### Interventions

Participants received five 20-min sessions per week over a period of 2 weeks (10 sessions in total). All the therapists have >5 years of clinical experience in the administration of systematic Tuina or routine rehabilitation training. After treatment, the two groups were provided with guidance on active qigong (Tuina practice) or rehabilitation training as applicable. The participants were not allowed to receive other treatments except for the basic antidepressant venlafaxine.

### Tuina therapy group

According to the traditional Chinese medicine theory, disorder of the Du meridian is closely related to the pathology of PSD, and its body surface markings start from the coccyx and runs along the spine up to the retro-occipital tuberosity (21). The acupuncture points along the Du meridian include Baihui [GV20], Dazhui [GV14], Zhiyang [GV9], Mingmen [GV4], and Changqiang [GV1]. For the Tuina therapy administered in the present study, the patient was instructed to lay on a massage table in a prone position. The therapist took 1 to 3 grams of the herbal ointment and applied it evenly to each acupoint. Using the thumb, the therapist pressed and kneaded the abovementioned points along the posterior midline of the spine for about 1–2 min. Bernhardth et al. (3) Thereafter, the index, middle, and ring fingers were used to rub along the line from Changqiang (GV1) to Dazhui (GV14) for 8–10 min. This was followed with quickly rubbing the palms back and forth along the Du meridian. After the treatment, the patients were instructed to undergo independent training on qigong (Tuina practice) - Baduanjin types 1 and 8. The schematic diagram of the Tuina intervention is shown in Supplementary Material.

### Routine rehabilitation control group

In China, routine rehabilitation is still the main the non-drug treatment for PSD. It is generally believed that the improvement of limb motor function can relieve depression. For the control group in the present study, routine rehabilitation was administered using the Bobath and Brunnstrom approaches. These therapies mainly include passive body positioning and bed training, sitting training, guided joint response and movement, guided separation movement, walking training, and daily life exercises.

### Outcome measures

All outcomes were assessed at two timepoints: baseline (after enrollment) and week 2 (intervention session).

#### Primary outcome

##### Hamilton depression scale score

The primary outcome measure was the change in HAMD score over the 2 weeks of intervention. We utilized the HAMD-24 version of the scale widely used by clinicians. The total score of the scale is 52 points, and the score is positively correlated to the degree of depression. A total score of 17 or higher indicates mild or major depressive disorder, a score of 0–7 indicates the absence of depressive symptoms, a score of 8–17 indicates mild depression, a score of 18–24 indicates moderate depression, and a score of 25–52 indicates severe depression (22).

#### Secondary outcomes

##### Fugl-meyer assessment

The Fugl-Meyer Assessment (FMA) scale is a tool commonly used for the clinical evaluation of limb motor function. It is mainly utilized to evaluate the exercise quality and performance of patients with different degrees of clinical impairment. The total score of the FMA scale is 100 points, with 33 items for the upper extremities and 17 items for the lower extremities. A total score <50 points (grade I) indicates the existence of severe movement disorders, a score of 50–84 points (grade II) indicates the existence of obvious movement disorders, a score of 85–95 points (grade III) indicates moderate dyskinesia, and a score of 96–99 points (grade IV) suggests mild dyskinesia (23).

##### Modified barthel index

The Modified Barthel Index (MBI) was used to evaluate 10 aspects of the activities of daily living (ADL) of the patients with PSD. The MBI is widely used to evaluate ADL function in clinical stroke rehabilitation (24).

##### Mini-mental state examination

The Mini-Mental State Examination (MMSE) was used to evaluate the cognitive ability of the patients with PSD. The total MMSE score is 30 points, distributed across seven aspects: 10 points for orientation, 5 points for attention and calculation, 3 points for memory, 3 points for recall, 3 points for language and visual space, 9 points. A total score ≥27 points indicates normal cognition, a score of 21–26 points indicates mild cognitive impairment, a score of 10–20 points indicates moderate cognitive impairment, and a score of 0–9 points indicates severe cognitive impairment.

### Magnetic resonance imaging data acquisition

The magnetic resonance imaging (MRI) scans of all the participants were taken between 16:30–19:30 to avoid bias from variations in the resting state of the brain at different times of the day. For the MRI procedure, the patient was asked to lay in a supine position with eyes closed and the head fixed in the coil. The patient wore earplugs to reduce noise interference and was instructed to remain awake, breathe calmly, and be as immobile as possible. Resting-state fMRI was performed using a 3.0T magnetic resonance equipment (MANGETOM Verio; Siemens AG, Germany). The scanning parameters used are as follows: repetition time = 3,000 ms, echo time = 30 ms, flip angle = 90°, slice thickness = 3 mm, field of view = 240 × 240 mm, acquisition matrix = 64 × 64, voxel resolution = 3.4 × 3.4 × 3.2 mm; number of slices = 43. A total of 240 TRs were scanned.

Data preprocessing was performed using Mricron software (https://www.nitrc.org/projects/mricron). The dcm2nii program in the package was converted to obtain resting-state and structural image data. The resting-state and structural image data of patients whose lesions are located on the left were transformed into left and right mirror images using the LR Flip function in the Mricron software to facilitate subsequent image analysis. Uncontrollable factors were excluded to further improve the quality of the data. Removal of unstable timepoints, temporal layer correction, head motion correction, origin correction, spatial registration, smoothing, de-linear trend, filtering, and covariate removal were performed.

### Safety evaluation

The side effects and safety events that occurred during the trial were recorded in a case report form, and prognosis and countermeasures are tracked.

### Sample size calculation

Our sample size was estimated using the HAMD score as the primary outcome. Based on a pre-trial of 2-weeks Tuina intervention compared with routine rehabilitation for PSD, there was a mean change between the baseline and post-treatments: 7.2 ± 7.1 (Tuina therapy group) vs. 3.1 ± 6.5 (routine rehabilitation control group) for the score of HAMD. Thus, assuming a significance level α of 0.05 and power (1-β) = 0.80, this trial require a minimum of 35 participants in each group to provide effective power to reject the null hypothesis. Considering the expected dropout rate of 20%, 42 participants will be recruited in each group.

### Statistical analyses

#### Analysis of clinical outcome data

SPSS software was used to conduct data statistics using the principle of intention-to-treat analysis. A two-sided significance level of 5% was adopted, and the corresponding 95% confidence interval (CI) was calculated. Statistical significance was set at *p* < 0.05. Baseline characteristics are reported as mean (standard deviation). A two-sided *t* test was used to adjust the baseline outcome values for the comparison of differences in mean changes between groups (post-intervention minus baseline). The chi-square test was used to compare dichotomy variable groups.

#### Analysis of resting-state data

##### Amplitude of low-frequency fluctuation

After preprocessing, the amplitude of low-frequency fluctuation (ALFF) of the brain regions were calculated using REST software (Beijing Normal University, http://www.restfmri.net). The time series of each voxel was transformed into a frequency range using Fourier transform, and the power spectrum of each voxel in the frequency band was obtained. Since the power at a given frequency is proportional to the square of the magnitude of that frequency component, the square root of the power spectrum was calculated at each frequency. Thereafter, the mean square root of the power spectrum in the different frequency bands (that is, the ALFF value) was calculated. Finally, the Z-transformation of the ALFF values for each participant was used for further between-group comparisons.

##### Analysis of functional connectivity

In this trial, brain regions related to emotional circuits were used as regions of interest (ROIs). REST software was used to analyze the anterior cingulate gyrus, amygdala, caudate nucleus, hippocampus, globus pallidus, par hippocampal gyrus, putamen, dorsal ROI-wise functional connectivity (FC) of 20 brain regions, such as the lateral prefrontal lobe, medial prefrontal lobe, and thalamus, to calculate the Pearson correlation between the time series of the two ROIs, extract the BOLD signal in the ROI, and obtain the average value of the ROI in the region. Regarding the time series, the FCMap.txt file output was obtained from the correlation analysis of other ROIs, and Fisher-Z transformation was performed to analyze the obtained values. The smoothed data were de-linear drifted and filtered (0.01–0.08 Hz), and covariates, including head movement parameters, globally-averaged signals, and white matter and cerebrospinal fluid signals, were removed. Thereafter, REST software was used to perform FC calculation, Fisher's Z transformation, and between-group statistical analysis.

## Results

**Figure 1:**
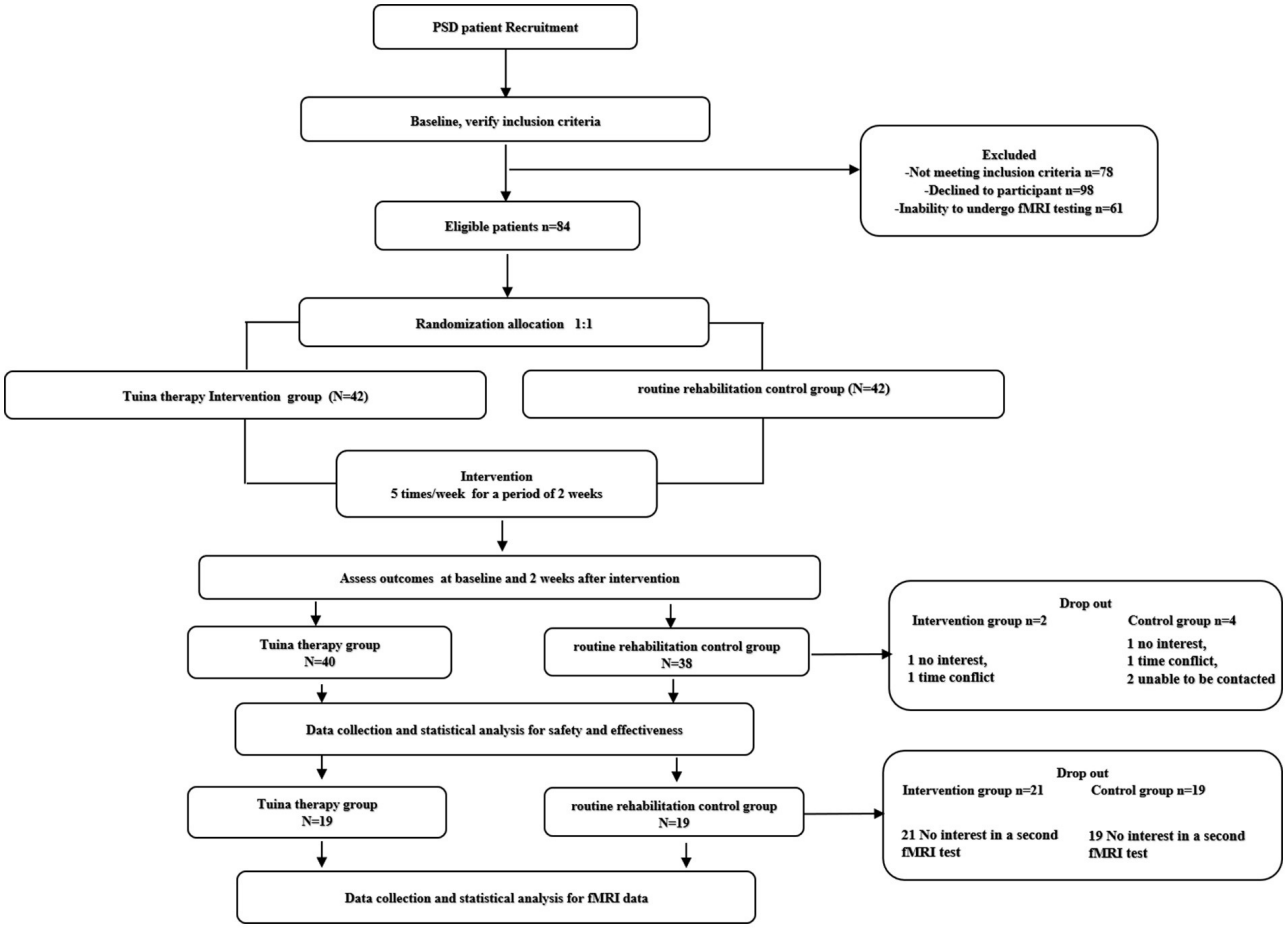
Flow chart of the research.

A total of 321 subjects with PSD were screened for inclusion into this trial. Of these, 84 (26.1%) were eventually included in the study. A total of 78 (92.8%) participants (40 [5.2%]) in the Tuina therapy group and 38 in the routine rehabilitation control group) completed clinical scale evaluation. The reasons for dropout included treatment intolerance, withdrawal from the trial, and adverse reactions that the investigator considered serious enough to discontinue the trial. The clinical data of all the participants who dropped out were imputed as means. The fMRI data of 19 participants in the two groups were collected and analyzed after 2 weeks; the remaining participants were excluded owing to invalid image acquisition or because they refused to undergo the test. No serious safety problems occurred during the trial. Except for two patients in the routine rehabilitation control group who had vertigo and discontinued the trial due to safety concerns, participants in the two groups (five in the Tuina group and four in the control group) showed some muscle soreness symptoms. However, the symptoms were relieved after rest, and they had no impact on the outcome measurements. There was no statistical difference between the baseline data of the two groups (Table 1). The flowchart of the research is shown in Figure 1.

**Table 1:** Demographic and clinical characteristics of the two groups.

**Characteristic**	**Tuina group**	**Routine rehabilitation group**	***P***
Age, years	62.40 ± 11.91	62.86 ± 12.91	0.43
Disease duration (months)	15.38 ± 9.29	13.14 ± 10.74	0.46
Sex: Male /Female(n)	25 (17)	18 (24)	0.5
Duration of education (year)	11.50 ± 3.96	12.48 ± 3.19	0.22
Current use of a monoamine oxidase inhibitor, n (%)	16(38%)	21(50%)	0.27
Use of tricyclic drugs, n (%)	13(30.9%)	10(23.8%)	0.63
Adverse events, n (%)	5(13.8%)	3(8.3%)	0.45
HAMD	16.14 ± 4.52	15.00 ± 4.27	0.47
FMA	57.88 ± 19.24	56.29 ± 18.94	0.7
MBI	61.19 ± 13.47	58.33 ± 15.91	0.38
MMSE	19.43 ± 5.19	18.93 ± 5.41	0.67

*HAMD, Hamilton Depression Scale; FMA, Fugl-Mayer scale; MBI, Modified Barthel index; MMSE, Minimum Mental State Examination.*

*Independent samples t-test and chi-square test were used for statistical analysis.*

*P < 0.05 was considered statistically significant*.

Regarding the primary outcome, the Tuina group showed significantly improved HAMD scores compared to the Routine rehabilitation group (5.85, [2.54, 9.16]). For the secondary outcomes, the Tuina group showed better MMSE scores than the Routine Rehabilitation group (1.97, [1.19, 2.76]); however, there were no significant differences between the other secondary outcomes of both groups (*P* > 0.05). After 2 weeks, both groups showed a significant decrease in HAMD scores compared to baseline. In addition, the Tuina group showed a significant decrease in MMSE score compared to baseline (2.35, [1.8, 2.9]); however, there were no significant differences in the MBI and FMA scores of the two group after the intervention (*P* > 0.05). The above results are shown in Table 2.

**Table 2:** Comparison of scale scores between and within groups*.

**Outcomes**	**Intervention phase**				**Within-group difference (week 2 minus week 0), mean (95% CI)#**		**Between-group difference (IG minus CG)**, **mean** **(95% CI)#**
	**Week 0**		**Week 2**				
	**Tuina group** **(*****n** =* **42)**	**Routine rehabilitation** **group (*****n** =* **42)**	**Tuina group** **(*****n** =* **40)**	**Routine rehabilitation** **group (*****n** =* **38)**	**Tuina group** **(*****n** =* **40)**	**Routine rehabilitation** **group (*****n** =* **38)**	
HAMD	16.14 ± 4.52	15.00 ± 4.27	9.90 ± 4.61	13.24 ± 4.36	2.9 (1.07–4.73)^&^	8.76 (6.83–10.69)^&^	5.85 (2.54–9.16)^&^
FMA	57.88 ± 19.24	56.29 ± 18.94	60.38 ± 17.50	59.38 ± 17.82	2.5 (−6.64–1.64)	2.71 (−8.32–2.89)	0.21 (−7.2–7.2)
MBI	61.19 ± 13.47	58.33 ± 15.91	62.50 ± 13.57	60.36 ± 13.94	1.3 (−4.4–1.78)	2.0 (−7–2.96)	0.71 (−5.16–6.62)
MMSE	19.43 ± 5.19	18.93 ± 5.41	21.79 ± 5.00	19.31 ± 5.07	2.35 (1.8–2.9)^&^	0.38 (−093–0.16)	1.97 (1.19–2.76)^&^

*HAMD, Hamilton Depression Scale; FMA, Fugl-Mayer scale; MBI, Modified Barthel index; MMSE, Minimum Mental State Examination.*

^*^Except where indicated otherwise, values are mean ± SD.

*95% CI: 95% confidence interval.*

*& P < 0.05.*

*# Adjusted for baseline values; two-sided T test was used for between-group comparisons*.

**Figure 2:**
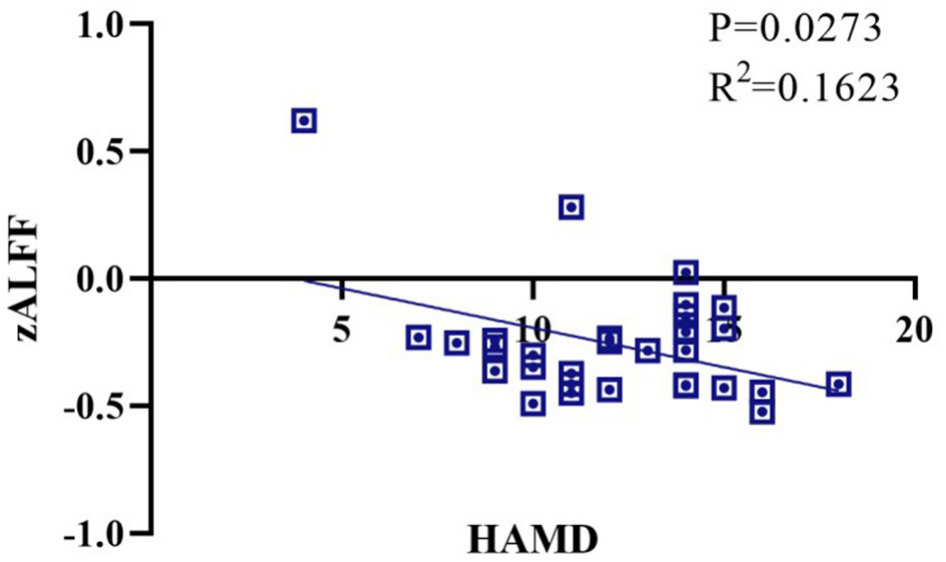
Correlation analysis of left hippocampal zALFF value and HAMD score.

The Tuina therapy group showed better zALFF values in the right caudate nucleus, right putamen, right insula, left superior temporal gyrus, right par hippocampal gyrus, lateral hippocampus, left middle temporal gyrus, left angular gyrus, and left thalamus than the Routine rehabilitation control group (Table 3 and Supplementary Material). The FC of the right amygdala and the right caudate nucleus, the left caudate nucleus and the left thalamus, the right caudate nucleus and right amygdala, right caudate nucleus and bilateral thalamus, left hippocampus and right thalamus, right hippocampus and right putamen, right par hippocampal gyrus and right putamen, right putamen nucleus and right thalamus were significantly different between the two groups (*P* <0.05) (Table 4 and Supplementary Material). The correlation analysis (Figure 2) of the ipsilateral hippocampal function imaging index zALFF value and HAMD score showed that zALFF value was significantly negatively correlated with HAMD score (*P* < 0.05).

**Table 3:** Comparison of the post-intervention zALFF values of the two groups.

				**MNI coordinates**		
Tuina therapy group> routine rehabilitation group	Region Label	Extent	t-value	x	y	z
	Caudate-R	34	4.488	15	21	9
	Putamen-R	106	4.357	33	3	6
	Insula-R	106	4.313	36	−15	15
	Temporal-Sup-L	22	4.234	−54	−9	6
	ParaHippocampal-R	99	3.980	33	−42	−9
	Hippocampus-R	99	3.422	33	−18	−21
	Temporal-Mid-L	29	3.657	−54	−60	21
	Angular-L	22	3.229	−51	−63	36
	Thalamus-L	14	2.572	−9	−21	12
Tuina therapy group < routine rehabilitation group	OFCant-L	33	−4.942	−24	63	−15
	OFCpost-L	21	−4.049	−30	21	−18
	Frontal-Sup-Medial-R	103	−3.948	3	30	60
	Frontal-Sup-2-R	103	−3.49	15	12	72
	Cingulate-Ant-R	59	−3.897	12	33	18
	Frontal-Sup-2-L	24	−3.87	−12	54	30
	Frontal-Mid-2-L	43	−3.84	−30	42	33
	Amygdala-R	13	−2.777	30	3	−24

*Right caudate nucleus, right putamen, right insula, left superior temporal gyrus, right parahippocamal gyrus, zALFF values of the right hippocampus, left middle temporal gyrus, left angular gyrus, and left thalamus were more dominant in the Tuina group than in the conventional rehabilitation group*.

**Table 4:** Functional connectivity of the brain regions.

	**AMYG-R**	**CAU-L**	**CAU-R**	**HIP-L**	**HIP-R**	**PHG-R**	**PUT-R**	**THA-L**	**THA-R**
AMYG-R	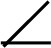		0.029						
CAU-L		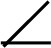						0.012	
CAU-R	0.029		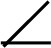					0.045	0.005
HIP-L				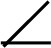					0.003
HIP-R					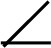		0.035		
PHG-R						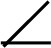	0.030		
PUT-R					0.035	0.030	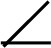		0.018
THA-L		0.012	0.045					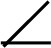	
THA-R			0.005	0.003			0.018		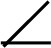

*Two-sample t-test of the functional connectivity correlation coefficient between the two groups.*

*P < 0.05 was considered statistically significant*.

## Discussion

In this study, we investigated the efficacy and fMRI outcomes of Tuina therapy in patients with PSD. The results showed that 2 weeks of Tuina therapy improved HAMD and MMSE scores better than routine rehabilitation. This is consistent with the results of previous studies on the clinical efficacy of Tuina for the treatment of mental disorders, such as depression and anxiety (25, 26). Although the participants in the present study have histories of taking small doses of antidepressants, the results of the present study provides clinical evidence of the efficacy of Tuina therapy alone, which is different from previous studies in which the interventions were mainly Tuina therapy combined with physical therapy (27, 28).

In the present study, there was no significant difference between the limb function activities and ADL recorded at baseline and at the 2-week timepoint. Some previous studies have shown that Tuina can improve limb function in patients with PSD; however, a longer treatment time and integrative therapy are required (17, 29). The Tuina group in the present study showed better zALFF values of the subcortical areas dominated by the hippocampus and thalamus than the Routine rehabilitation control group. In addition, the Tuina group showed significantly enhanced excitability in these regions. The statistical results of FC between the nucleus and the right thalamus were significantly different between the Tuina and control groups. In the Tuina group, the FC between the hippocampus and the thalamus was the most significant. Correlation analysis showed that the two were negatively correlated. This confirmed that the mechanism of Tuina intervention for PSD is closely related to the emotional circuits in the hippocampus and thalamus. This is consistent with the findings of previous related studies, which indicate that Tuina therapy can activate the hippocampus and insula, and promote central remodeling of the limbic system (30, 31). Tuina therapy has less side effects than pharmacotherapy, which encourages patient compliance and enables patients to have a good treatment experience. The mechanism of the effect of Tuina on the Du meridian may be related to mechanical stimulation of the tactile receptors around the spine to receive mechanical signals and convert them into electrical signals. These signals are then transmitted to neurons, which transmit them to the cerebral cortex through neurotransmitters in the upward sensory conduction pathway, leading to neural remodeling in the brain (32). Different types of Tuina therapy have different effects on the human corticospinal tract. This may be part of the peripheral-central mechanism of the effect of Tuina therapy. At present, there are only a few relevant studies on the mechanism of Tuina therapy. However, the results of some animal studies have shown that the analgesic mechanism of Tuina may be related to the inhibition of the upward conduction of pain by stimulating receptors on the skin (33, 34). Recently, some high-quality research showed that the molecular and neural mechanisms by which a pleasant touch similar to the Tuina effect can reduce stress may be related to the PROK2 neuropeptide encoding pathway and transmission to PROKR2 neurons in the spinal cord (35).

The results of the present study were biased because physical therapy could not be blinded. In addition, although the therapists who administered the interventions are uniformly trained, there may have been slight differences in the administration of the therapies. Future studies are needed to confirm the effect of this variable on the outcomes of the intervention. Finally, there were individual differences in the quality of the completion of active exercise between the two groups, which, although supervised during the trial, is also a potential variable. Therefore, the conclusions were carefully defined.

## Conclusion

This research demonstrated the superior effect of two-week Tuina therapy on depression and mental health compared with routine rehabilitation therapy. However, the results showed that Tuina therapy has no significant effect on physical activity. The results also showed that Tuina intervention could enhance the FC of the hippocampus and thalamus in patients with PSD. In addition, we noted a linear correlation between the FC of the hippocampus and thalamus and the improvement of clinical depression, which indicates that the Papez circuit with the hippocampus and thalamus as its core may become a new area of interest in research.

## Data availability statement

The original contributions presented in the study are included in the article/Supplementary Material, further inquiries can be directed to the corresponding author/s.

## Ethics statement

The studies involving human participants were reviewed and approved by Ethics Committee of Yueyang Hospital of Integrated Traditional Chinese and Western Medicine affiliated to Shanghai University of Traditional Chinese Medicine. The patients/participants provided their written informed consent to participate in this study.

## Author contributions

JT planned the study protocol and drafted the manuscript. SZ participated in the critical revision of the manuscript. LK carried out the Tuina and control interventions of patients. JW and QG recruited and screened eligible participants in the inpatient and outpatient department. MF managed the study and participated in the critical revision of the manuscript. CY participated in designing the outcome measurements and assessing the outcomes. QZ participated in designing the trial and helped to prepare the manuscript. CS provided fMRI devices to meet the evaluation requirements of the experiment. All the authors have read and approved the final manuscript.

## Funding

This study was supported by the Shanghai Municipal Health Commission Clinical Industry Special Project—A randomized controlled clinical study on PSD intervention by Tuina with herbal ointment therapy (2019404), the Key project of National Natural Science Foundation of China (82030121), Shanghai Super Postdoctoral Incentive Program (2021316), Shanghai Science and Technology Commission Qixing “Flying Project” (22YF1449000), Shanghai Pujiang Program (21PJD071), Talent development program in Shanghai (2019048), and Shanghai University of Traditional Chinese Medicine Budget Project (2021LK101). The funders had no role in the design of the study, the analysis, collection, interpretation of the data, the writing of the manuscript, and the decision to publish it.

## Conflict of interest

The authors declare that the research was conducted in the absence of any commercial or financial relationships that could be construed as a potential conflict of interest.

## Publisher's note

All claims expressed in this article are solely those of the authors and do not necessarily represent those of their affiliated organizations, or those of the publisher, the editors and the reviewers. Any product that may be evaluated in this article, or claim that may be made by its manufacturer, is not guaranteed or endorsed by the publisher.
