# Supplementary material for: Effectiveness and functional magnetic resonance imaging outcomes of Tuina therapy in patients with post-stroke depression: A randomized controlled trial
Source: Front Psychiatry. 2022 Jun 30;13:923721. doi: 10.3389/fpsyt.2022.923721 (PMC9281445; doi:10.3389/fpsyt.2022.923721)
Supplement: Supplementary file 1 [file Data_Sheet_1.docx]

Supplementary Material

## Functional magnetic resonance imaging


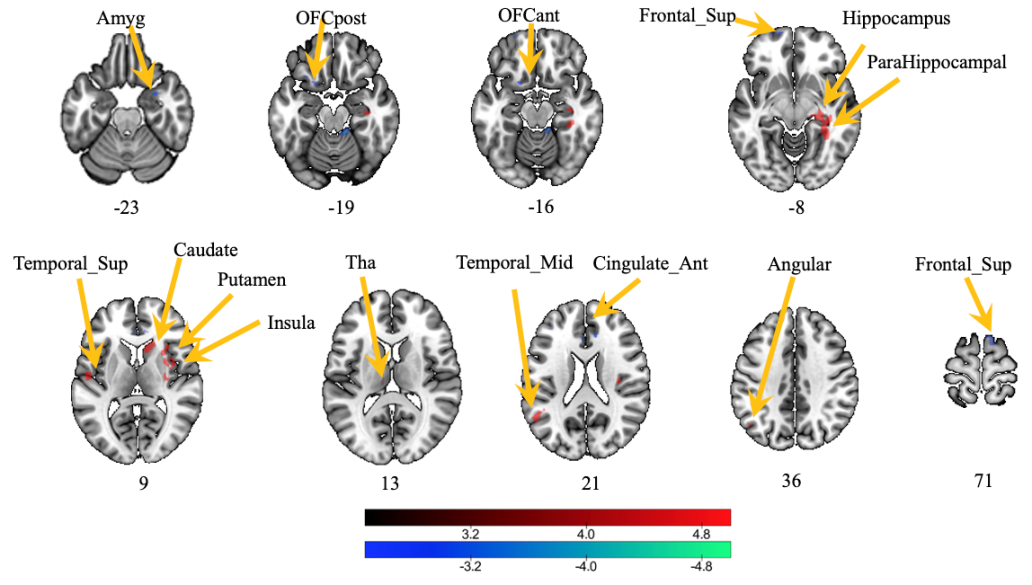


**Supplementary Figure 1.** Low frequency oscillation amplitude (zALFF) was compared between the two groups after treatment. Note: red indicates that the zALFF of the experimental group is larger than that of the control group, whereas blue-green indicates that the zALFF of the experimental group is smaller than that of the control group.


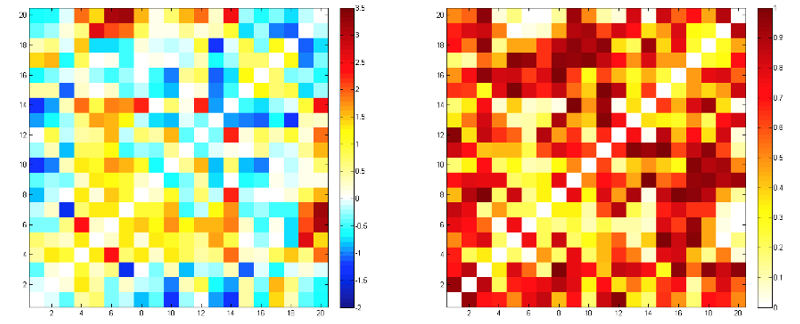


**Supplementary Figure 2.** T-value plot (A) and P-value plot (B) of the functional connectivity (FC) of emotion-related brain regions after treatment. Figure A is the t-value diagram of the inter-group comparison of the FC correlation coefficients for each pair of the brain regions (red and yellow represent positive values, whereas blue represents negative values). Figure B is the inter-group comparison of the P values of the FC correlation coefficients for each pair of the brain regions (the more yellow the color is, the smaller the P values are).


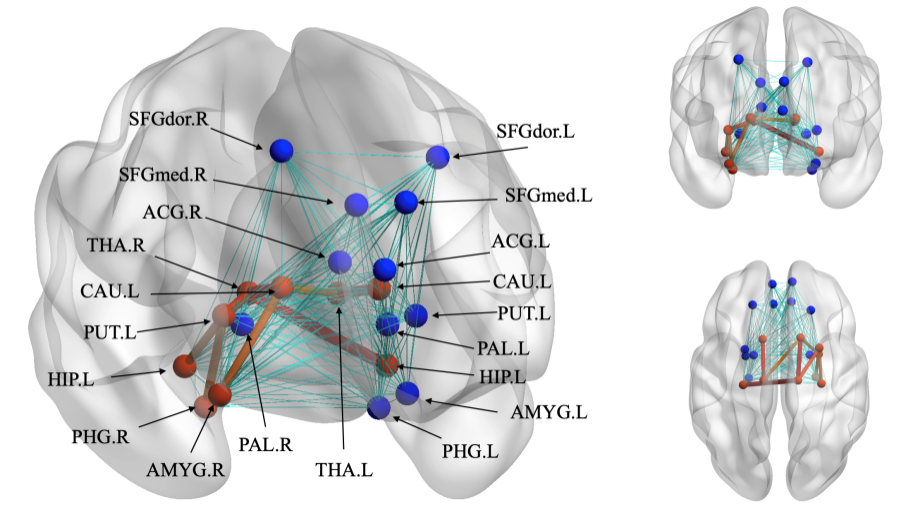


**Supplementary Figure 3.** Comparison of the functional connections of emotion-related brain regions between the two groups after treatment Note: red indicates the functional connections that are significantly different between the two groups (greater in the experimental group than in the control group).


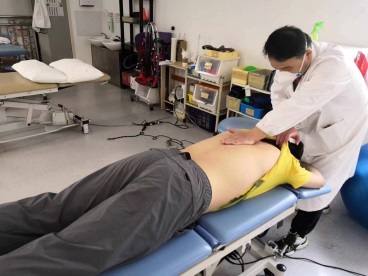
 （A）
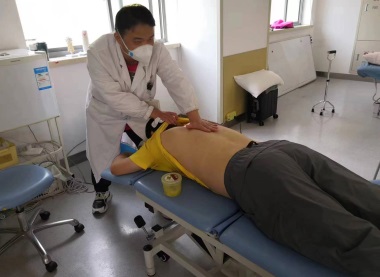
 (B)
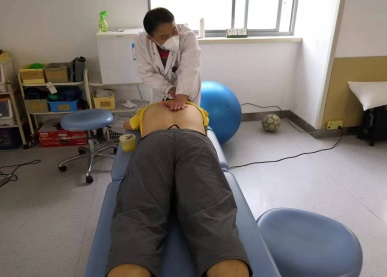
(C)


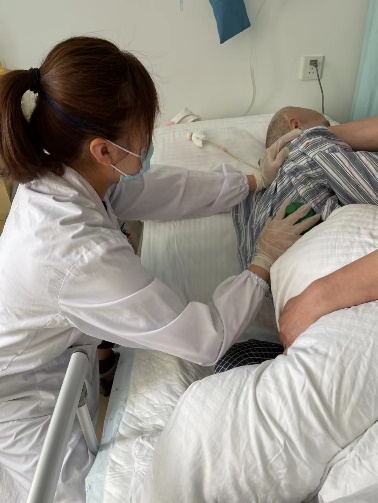
 (D)
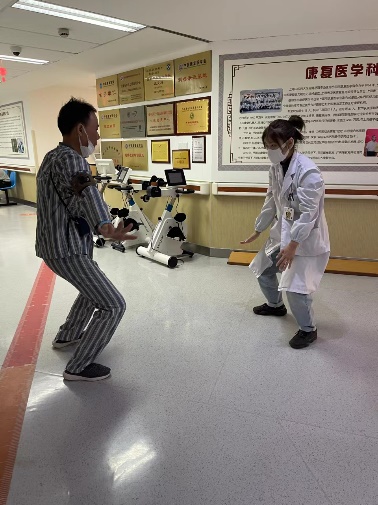
 (E)
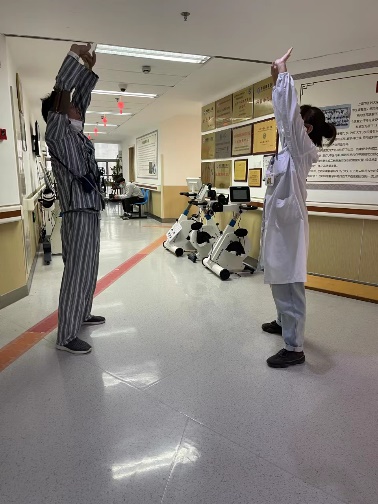
 (F)

**Supplementary Figure 4.** Some photographs of the intervention procedure. A-D: “Tong Du” manipulation therapy, E-F: Type1and 8 in Baduanjin Qigong.

**Supplementary Table 1**. Tuina intervention program.

| Tuina interventions | Details of Tuina interventions | | | | | |
| --- | --- | --- | --- | --- | --- | --- |
| “Tong Du”  head and face manipulation | Top-of –the –head Tuina | Knead “Baihui” point |  |  |  |  |
| “Tong Du "  Back of the waist | Knead “Mingmen”  point | Back stroke | Knead the middle of the spine | Rubbing the lumbosacral part |  |  |
| Dialectical Tuina | Depression of the Qi in the liver | Transformation of depressed Qi into fire | Stagnation of phlegm Qi | Insufficiency of heart and spleen | Insufficiency of heart to mentally confused | Fire excess from yin deficiency |
|  | 1.Wide chest method  2.Massage ribs | Push into the abdomen | Knead “Tiantu”  point | 1.Massage the upper abdomen  2. Massage around the navel | 1.Rub the sides of the head  2. Knead behind the occipital | 1.Massage the waist horizontally  2.Knead the “Shenmen”  point |
| Qigong  (Tuina practice) | Holding the hands high with palms up to regulate the internal organs | Raising and lowering the heels to cure diseases |  |  |  |  |
